# Frailty as a Predictor of COVID-19 Mortality in the South Indian Population: An Observational Study

**DOI:** 10.7759/cureus.70820

**Published:** 2024-10-04

**Authors:** Niveda Srivatsa, Nirmala Devi Chandrasekaran, Mohammed Suhail Tazeem, Priya Vijayakumar

**Affiliations:** 1 Geriatrics, SRM Medical College Hospital and Research Centre, SRM Institute of Science and Technology (SRMIST), Chennai, IND; 2 General Medicine, SRM Medical College Hospital and Research Centre, SRM Institute of Science and Technology (SRMIST), Chennai, IND; 3 Geriatrics, Amrita Hospital and Research Institute, Kochi, IND

**Keywords:** clinical frailty, covid-19 india, elderly patients, mortality predictors, sars-cov-2

## Abstract

Background

Frailty is a clinical syndrome characterized by diminished strength, endurance, and physiological function that significantly increases vulnerability to adverse health outcomes, including infections. In the context of COVID-19, frailty has emerged as a critical risk factor for severe disease, complications, and mortality, particularly in older adults. The severity and fatality rates among the geriatric group were notably high, as the virus's pathogenesis, marked by prolonged inflammation, contributed to increased morbidity and mortality in this age group. The study was conducted to explore the role of frailty in influencing mortality among the elderly affected by COVID-19.

Objective

The objective of this study was to identify the association between frailty and mortality in COVID-19-affected elderly patients.

Methods

We conducted a prospective observational study among elderly patients who tested positive for COVID-19 and received treatment in a tertiary care hospital. Data were collected from 250 patients from March 2021 to December 2021. Lab parameters, the necessity for mechanical ventilation, the need for oxygen use, and the number of days of hospital stay were recorded. The Clinical Frailty Score (CFS) was used to evaluate frailty. The chi-square test with Fisher’s exact test was used to assess the association between frailty and mortality in the data set. Multivariate binary logistic regression was employed to identify the most significant predictors of mortality.

Results

Among the 250 patients, 159 (63.6%) survived and were discharged, while 91 (36.4%) succumbed to the illness. Fifty-eight patients were not identified as frail, and there were no deaths in the group. On the contrary, among the 192 COVID-positive patients who were identified as frail, 91 (47.4%) patients died, and 101 (52.6%) patients were alive. This depicted the association between frailty and mortality in COVID-19 geriatric patients. While assessing comorbidities, malignancy (53.3%, p-value = 0.009) and chronic kidney disease (CKD) (43.3%) had a significant association with mortality. Symptoms like fever (43.6%), dyspnea (68.6%), myalgia (20%), and altered sensorium (84%) showed a strong correlation with mortality (p<0.001). Frailty was a significant predictor of mortality, with 47.4% of frail patients not surviving (p<0.001). Biochemical markers including leukocytosis (64.8%), neutrophilia (65.3%), eosinopenia (66.9%), anemia (57.8%), hypoalbuminemia (63.5%), hypoproteinemia (70.1%), elevated alanine aminotransferase (ALT) (66%), aspartate aminotransferase (AST) (65.2%), alkaline phosphatase (ALP) (67.5%), elevated creatinine (68.9%), hypernatremia (100%), hyperkalemia (80%), and elevated D-dimer (44.7%) were all significantly linked to mortality. Additionally, patients requiring oxygen (65%), ventilation (96.8%), or bilevel positive airway pressure (BiPAP) (77.8%) had higher mortality rates. A shorter length of hospital stay was also associated with increased mortality (24%).

Conclusion

Frailty, combined with certain comorbidities such as cancer and CKD, along with various clinical and biochemical markers, played a significant role in predicting mortality among geriatric COVID-19 patients. Incorporating frailty assessments into routine evaluations for elderly COVID-19 survivors could be beneficial. Early detection and focused management of these high-risk factors are essential for improving outcomes in frail patients within tertiary care settings.

## Introduction

The SARS-CoV-2 virus causes COVID-19, which spreads via respiratory droplets and has led to a global pandemic that has caused millions of deaths worldwide. Due to lowered immunity, infection is particularly common among older people [1]. The disease can present in a variety of ways, ranging from asymptomatic cases to severe symptoms, and it can be fatal [2]. Frailty is a multifactorial syndrome that is characterized by decreased strength, endurance, and physiologic function, increasing an individual's vulnerability to heightened dependency and/or death. While the risk of frailty rises with age, it can also affect younger individuals. There is strong evidence that frailty is linked to poorer outcomes for hospitalized patients, including both medical and surgical admissions, as well as those requiring intensive care. This growing recognition has led to frailty being used as a key indicator for resource distribution, decision-making pathways, and shared care discussions [3]. Definitive predictors of poor clinical outcomes remain uncertain. Finding reliable mortality predictors in critically ill patients is complex, given the range of critical conditions like acute respiratory distress syndrome, secondary infections, shock, and acute heart and kidney injuries. Laboratory parameters are particularly important as they can indicate mechanisms of disease progression and offer valuable insights into possible therapeutic interventions [4]. Literature has suggested biochemical markers are often elevated in patients as COVID-19 results in significant organ damage, mainly to the heart, liver, and kidneys. Survivors had significantly lower levels of lactate dehydrogenase (LDH), creatine kinase (CK), alanine aminotransferase (ALT), aspartate aminotransferase (AST), creatinine, and N-terminal pro-brain natriuretic peptide [5].

## Materials and methods

Selection and description of participants

A prospective observational study was conducted in Amrita Hospital, Amrita Institute of Medical Sciences (AIMS), a tertiary care hospital in, Kochi, India from March 2021 to December 2021. We analyzed the data of 250 patients who met the inclusion criteria of being over 60 years of age and diagnosed with COVID-19, all of whom were admitted to a tertiary care center in South India. Subjects unwilling to participate were excluded. Statistical tests were performed accordingly. The study was initiated after obtaining approval from the Institutional Ethics Committee of Amrita Institute of Medical Sciences, Kochi, India (approval number: ECASM-AIMS-2021-338, dated 28/9/2021). Patient details, including sociodemographic data, comorbidities, symptoms, vitals, and lab parameters, were collected. Frailty was assessed using the Clinical Frailty Score (CFS), which evaluates specific domains including comorbidity, function, and cognition to generate a frailty score ranging from one to nine: Level 1: very fit, Level 2: well, Level 3: managing well, Level 4: apparently vulnerable, Level 5: mildly frail, Level 6: moderately frail, Level 7: severely frail, Level 8: very severely frail, and Level 9: terminally ill [6]. According to CFS, patients with a score of less than four are considered not frail, while those with a score of four or above are considered frail. Oxygen requirement and the need for bilevel positive airway pressure (BiPAP) or mechanical ventilation were also assessed. The outcome of each patient in the hospital, including the total number of in-hospital days, was also recorded.

Statistical analysis

Statistical analysis was done using IBM SPSS Statistics software, version 20.0 (IBM Corp., Armonk, NY). To test the statistical significance of differences in the proportion of comorbidities, clinical symptoms, vital signs, biochemical parameters, oxygen requirement, use of mechanical ventilation, and length of hospital stay, the chi-square test with Fisher’s exact test was applied. Multivariate binary logistic regression was used to identify the most significant predictors of mortality. Statistical significance was set at a p-value <0.05.

## Results

Baseline characteristics

Among the 250 patients, 159 (63.6%) survived and were discharged, while 91 (36.4%) succumbed to the illness. Patients were categorized into three age groups: 60-70 years, 71-80 years, and ≥81 years. In the 60-70 years age group, there were 134 patients (53.6%); in the 71-80 years age group, there were 78 patients (31.2%); and in the ≥81 years age group, there were 38 patients (15.2%). Of the 250 patients, 159 (63.6%) were male, and 91 (36.4%) were female.

Association with mortality

The major observations in the two groups are shown in Table 1. There were 192 patients with frailty, of which 101 (52.6%) survived, while the remaining 91 (47.4%) died during the course of the hospital stay. Fifty-eight patients were not identified as frail, and there were no deaths in the group. In contrast, among the 192 COVID-positive patients who were identified as frail, 91 (47.4%) patients died, and 101 (52.6%) patients were alive. The association of mortality between groups was statistically significant, with a p-value of <0.001. This clearly demonstrated the link between frailty and mortality and its adverse clinical outcomes in COVID-19 geriatric patients.

**Table 1:** Comparison of demographic, clinical, and biochemical characteristics between alive and dead COVID-19 patients from chi-square and Fisher’s exact tests

Variable	Alive, n (%)	Dead, n (%)	p-value
Age, in years			0.883
60-70	87 (64%)	47 (35%)	
71-80	48 (61.5%)	30 (38.5%)	
>81	24 (63%)	14 (36%)	
Gender			0.759
Male	100 (62.9%)	59 (37.1%)	
Female	59 (64.8%)	32 (35.2%)	
Comorbidities			
Systemic hypertension	105 (64.4%)	58 (35.6%)	0.713
Diabetes mellitus	89 (59.7%)	60 (40.3%)	0.123
Coronary artery disease	48 (58.5%)	34 (41.5%)	0.245
Dyslipidemia	66 (68%)	31 (32%)	0.245
Chronic kidney disease	19 (41.3%)	27 (58.7%)	0.001
Malignancy	21 (46.7%)	24 (53.3%)	0.009
Cerebrovascular accident	14 (48.3%)	15 (51.7%)	0.068
Bronchial asthma	8 (47.1%)	9 (52.9%)	0.142
Chronic liver disease	9 (50%)	9 (50%)	0.213
Hypothyroidism	22 (68.8%)	10 (31.3%)	0.517
Obstructive sleep apnea	1 (20%)	4 (80%)	0.060
Parkinson’s disease	4 (50%)	4 (50%)	0.467
Benign prostatic hyperplasia	18 (64.3%)	10 (35.7%)	0.936
Chronic obstructive pulmonary disease	11 (52.6%)	10 (47.6%)	0.264
Symptoms			
Fever	102 (56.4%)	79 (43.6%)	< 0.001
Cough	95 (59.7%)	64 (40.3%)	0.094
Dyspnea	48 (38.4%)	77 (68.6%)	< 0.001
Anosmia	22 (95.7%)	1 (4.3%)	< 0.001
Myalgia	44 (80%)	11 (20%)	0.004
Fatigue	48 (75%)	16 (25%)	0.002
Loose stools	16 (76.2%)	5 (26.8%)	0.210
Altered sensorium	4 (16%)	21 (84%)	< 0.001
Rhinitis	24 (96%)	1 (4%)	< 0.001
Asymptomatic	14 (100%)	0	0.003
Frailty			
No	58 (100%)	0 (0%)	< 0.001
Yes	101 (52.6%)	91 (47.4%)	< 0.001
Oxygen requirement			
No	110 (100%)	0 (0%)	< 0.001
Yes	49 (35%)	91 (65%)	< 0.001
Bilevel positive airway pressure requirement			
No	135 (95.1%)	7 (4.9%)	< 0.001
Yes	24 (22.1%)	84 (77.8%)	< 0.001
Ventilator requirement			
No	157 (83.5%)	31 (16.5%)	< 0.001
Yes	2 (3.2%)	60 (96.8%)	< 0.001
Length of stay in the hospital			
0-7 days	95 (76%)	30 (24%)	< 0.001
8-14 days	34 (56.7%)	26 (43.3%)	< 0.001
15-21 days	17 (56.7%)	13 (43.3%)	< 0.001
21-50 days	13 (37.1%)	22 (62.9%)	< 0.001

Among the various comorbidities, malignancy (p = 0.009) and chronic kidney disease (CKD) (p = 0.001) were significantly associated with mortality. Several symptoms also showed significant associations with mortality, including fever (p < 0.001), dyspnea (p < 0.001), anosmia (p < 0.001), myalgia (p = 0.004), fatigue (p = 0.028), altered sensorium (p < 0.001), and rhinitis (p < 0.001). Vital parameters significantly correlated with mortality were hypotension (p < 0.001), elevated blood pressure (p < 0.001), bradycardia (p < 0.001), tachycardia (p < 0.001), desaturation (p < 0.001), bradypnea (p < 0.001), and tachypnoea (p < 0.001). Oxygen requirement (p < 0.001), ventilation requirement (p < 0.001), and BiPAP usage (p < 0.001) were also significantly associated with mortality. The various laboratory parameters analyzed are given in Table 2. 

**Table 2:** Comparison of biochemical parameters between alive and dead COVID-19 patients using Chi-square and Fisher’s exact test WBC: white blood cell; MCV: mean corpuscular volume; CRP: C-reactive protein; AST: aspartate aminotransferase; ALT: alanine aminotransferase; ALP: alkaline phosphatase; LDH: lactate dehydrogenase

Biochemical Parameters	Reference range	n	Alive, n (%)	Dead, n (%)	p-value
WBC					
Leukocytosis	>11,000 per microliter	108	38 (35.2%)	70 (64.8%)	<0.001
Normal WBC	4,000-11,000 per microliter	132	115 (87.5%)	17 (12.9%)	<0.001
Leukopenia	<4000 per microliter	10	6 (60%)	4 (40%)	<0.001
Neutrophils					
Neutrophilia	>80%	121	42 (34.7%)	79 (65.3%)	<0.001
Normal neutrophils	40-80%	99	90 (90.9%)	9 (9%)	<0.001
Neutropenia	<40%	30	27 (90%)	3 (10%)	<0.001
Eosinophils					
Eosinopenia	<1%	121	40 (33.1%)	81 (66.9%)	<0.001
Normal eosinophils	1-4%	129	119 (92.2%)	10 (7.8%)	<0.001
Hemoglobin					
Normal hemoglobin	12-15 g/dl	124	100 (80.6%)	24 (19.4%)	<0.001
Anemia	<12 g/dl	123	58 (47.2%)	65 (57.8%)	<0.001
Polycythemia	>15 g/dl	3	1 (33.2%)	2 (66.8%)	<0.001
MCV					0.05
Normal MCV	83-101 fL	153	106 (69.3%)	47 (30.7%)	0.05
Macrocytic	>101 fL	5	2 (40%)	3 (60%)	0.05
Microcytic	<83 fL	92	51 (55.4%)	41 (44.6%)	0.05
CRP					0.087
CRP normal	8-10 mg/l	5	5 (100%)	0 (0%)	0.087
Elevated CRP	>10 mg/l	245	154 (62.9%)	91 (37.1%)	0.087
Albumin					<0.001
Hypoalbuminemia	<3.5 g/dl	126	46 (36.5%)	80 (63.5%)	<0.001
Normal albumin	3.5-5.5 g/dl	124	113 (91.1%)	11 (8.9%)	<0.001
Globulin					0.004
Normal globulin	2-3.5 g/dl	205	140 (68.3%)	65 (38.7%)	0.004
Hypoglobulinemia	<2 g/dl	22	9 (40.9%)	13 (59.1%)	0.004
Hyperglobulinemia	>3.5 g/dl	23	10 (43.5%)	13 (53.5%)	0.004
Protein					<0.001
Normal protein	6.0-8.3 g/dl	167	132 (82%)	29 (18%)	<0.001
Hypoproteinemia	<6.0 g/dl	87	26 (29.9%)	61 (70.1%)	<0.001
Hyperproteinemia	- >8.3 g/dl	2	1 (50%)	1 (50%)	<0.001
AST					
AST normal	8-33 U/L	183	135 (73.8%)	48 (26.2%)	<0.001
AST elevated	>33 U/L	67	24 (35.8%)	43 (65.2%)	<0.001
ALT					
ALT normal	7-56 U/L	197	141 (71.6%)	56 (28.4%)	<0.001
ALT elevated	>56 U/L	53	18 (34%)	35 (66%)	<0.001
ALP					
ALP normal	44-147 IU/L	210	146 (69.5%)	64 (30.5%)	<0.001
ALP elevated	>147 IU/L	40	13 (32.5%)	27 (67.5%)	<0.001
Creatinine					
Creatinine normal	0.7-1.3 mg/dl	153	122 (79.7%)	31 (20.3%)	<0.001
Elevated creatinine	>1.3 mg/dl	97	37 (38.1%)	60 (68.9%)	<0.001
Sodium					
Normal sodium	135-145 meq/L	146	108 (74%)	38 (26%)	<0.001
Hyponatremia	<135 meq/L	94	51 (54.3%)	43 (45.7%)	<0.001
Hypernatremia	>145 meq/L	10	0 (0)	10 (100%)	<0.001
Potassium					
Normal potassium	3.5-5.2 mEq/L	197	145 (73.6%)	52 (23.4%)	<0.001
Hypokalemia	< 3.5 mEq/L	33	10 (30.3%)	23 (69.7%)	<0.001
Hyperkalemia	>5.2 mEq/L	20	4 (20%)	16 (80%)	<0.001
D-dimer					
Normal D-dimer	<0.50 ng/ml	61	57 (93.4%)	4 (6.6%)	<0.001
Elevated D-dimer	>0.50 ng/ml	188	102 (54.3%)	86 (44.7%)	<0.001
Ferritin					
Normal ferritin	13-150 ng/ml	96	91 (94.6%)	5 (5.2%)	<0.001
Elevated ferritin	>150 ng/ml	141	58 (48.1%)	83 (58.9%)	<0.001
LDH					
Normal LDH	105-233 units/L	113	109 (96.5%)	4 (3.5%)	<0.001
Elevated LDH	>233 units/L	115	34 (29.6%)	81 (70.4%)	<0.001

Table 3 presents the multivariate analysis of significant risk factors for mortality in COVID-19 patients. Eosinopenia, when compared to normal eosinophil levels, is associated with a 3.373-times increased risk of mortality (odds ratio (OR) 3.373, p = 0.024, CI 1.177-9.669). Hypoalbuminemia significantly elevates the risk of death, with a 7.228-fold increase compared to normal albumin levels (OR 7.228, p < 0.001, CI 2.391-21.852). Elevated AST levels are linked to a 3.531-times higher mortality risk compared to normal AST levels (OR 3.531, p = 0.018, CI 1.246-10.005). Elevated creatinine is associated with a 2.258-times increased risk of mortality compared to normal creatinine levels (OR 2.258, p = 0.099, CI 0.858-5.942). The need for BiPAP significantly raises the mortality risk by 68.227 times compared to not requiring BiPAP (OR 68.227, p < 0.001, CI 16.813-276.856). Lastly, a shorter hospital stay of 0-7 days is associated with a 6.531-times increased risk of mortality compared to a longer stay of 21-50 days (OR 6.531, p = 0.028, CI 1.225-34.805).

**Table 3:** Results of multivariate analysis of significant risk factors for mortality in COVID-19 patients OR: odds ratio; CI: confidence interval; AST: aspartate aminotransferase; BiPAP: bilevel positive airway pressure

Factors	p-value	OR	CI for OR
Eosinopenia	0.024	3.373	1.177-9.669
Hypoalbuminemia	<0.001	7.228	2.391-21.852
Elevated AST	0.018	3.531	1.246-10.005
Elevated creatinine	0.099	2.258	0.858-5.942
BiPAP requirement	<0.001	68.227	16.813-276.856
0-7 days of stay	0.028	6.531	1.225-34.807
8-14 days of stay	0.147	2.834	0.694-11.563
15-21 days of stay	0.68	0.751	0.193-2.919

## Discussion

The study analysis revealed that certain comorbidities, such as CKD and malignancy, were associated with a higher risk of mortality. Among the symptoms, fever, dyspnea, altered sensorium, anosmia, myalgia, and fatigue were significantly associated with increased mortality rates. Vitals such as hypotension, elevated blood pressure, bradycardia as well as tachycardia, desaturation, bradypnea, and tachypnoea were also linked to an increased risk of death. Laboratory parameters that had a significant correlation with mortality included leukocytosis, neutrophilia, eosinopenia, anemia, hypoalbuminemia, elevated liver enzymes, elevated creatinine, hypernatremia, hyponatremia, hyperkalemia, elevated D-dimer, and raised ferritin levels. Additionally, the need for oxygen, non-invasive ventilation with BiPAP, and mechanical ventilation is attributed to an increased risk of mortality.

Rottler et al. conducted a meta-analysis of 42 cohort studies in the USA in 2022, primarily using the CFS, and found that frailty significantly increased mortality risk. Patients with CFS four to nine had a 3.12 times higher chance of death compared to those with CFS one to three (non-frail), with consistent results across both studies done in the United Kingdom (UK) and not done in the UK. Age did not impact outcomes, as studies with and without age restrictions showed similar mortality risks. Subgroup analyses by country (UK studies vs. non-UK studies), age (>65 years vs. no age restriction), and mortality (in-hospital vs. 30-day mortality) confirmed higher mortality in frail patients invariably. Non-survivors generally had higher frailty scores than survivors, with no influential study altering these results. The meta-analysis concluded that long-term outcomes in survivors should be followed up to determine whether frailty-based patient management is necessary for post-COVID sequelae [7]. Sablerolles et al. in 2021 conducted a multicenter retrospective study in 63 hospitals in 11 countries in Europe between March 30 and July 15, 2020; the total sample size was 2,434; and found a significantly higher risk of mortality in frail and mildly frail patients compared to fit patients (p < 0.0001, OR: 2.71 (95% CI 2.04-3.60)). The study findings indicate that the CFS score is an effective risk marker for hospital mortality among adult patients with COVID-19. Frail patients (CFS scores six to nine) and mildly frail patients (CFS scores four to five) aged 65 years and older exhibited significantly higher hospital mortality risks compared to fit patients (CFS scores one to three) [8]. In this study, frailty was similarly linked to mortality, contributing 47.4%, with a p-value of <0.001. In another meta-analysis by Thakur et al., among the Asian population, patients with known chronic kidney disease had a higher prevalence of disease severity (51%). In the same study, severe cases of COVID-19 were more prevalent among individuals with cardiovascular disease (44%), cerebrovascular accident (43%), Type 2 diabetes mellitus (42%), and respiratory disease (42%), with slightly lower rates observed in those with hypertension (39%) and malignancy (37%). The lowest percentage of disease severity (23%) was seen in liver disease patients [9]. In an epidemiological study done in China by Fang et al. in 2020, it was found that COVID-19 patients with chronic kidney disease had a relative risk of 7.1, and those with a history of stroke had a relative risk of 5.2; both had a significant risk of mortality [10]. However, Klein et al. in 2021 at Brigham found no association between cancer patients (27.8%) and non-cancer patients (25.6%) in relation to mortality [11]. In the present study, there was a strong link between CKD (58.7%, p = 0.001) and malignancy (53.3%, p = 0.009) and mortality among older adults with COVID-19.

A retrospective cohort study by Huang et al. conducted in Hubei, China, in 2020 found that 13.2% of patients with hypoalbuminemia died, compared to 1% of individuals with normal albumin levels (p < 0.001) [12]. In our study, there was a significant association of hypoalbuminemia with mortality (63.5%, OR 7.228, p <0.001, CI 2.391-21.852). Arnau et al. conducted a study in 2021 that concluded that the non-survivors group had a notably higher proportion of patients with serum albumin levels of ≤3 g/dL compared to survivors (11 (13%) vs. 14 (6%); p = 0.035) and had more severe disease. Furthermore, older adults with severe hypoalbuminemia had significantly increased death rates compared to their counterparts with normal serum albumin levels (84 (21%) vs. 11 (44%); p < 0.001) [13]. A retrospective study conducted by Kumar et al. in the USA in 2020 showed similar results, with 56% of patients found to have transaminitis. In the univariate analysis investigating the relationship between death and the presence of transaminitis, those with transaminitis had 2.9 times higher chances of death than those without transaminitis (p = 0.03) [14]. Our study also showed an association of transaminitis with adverse outcomes like death (AST elevation 65.2%, ALT elevation 66%, p <0.001).

Yan et al., in a 2020 retrospective study, showed that eosinopenia was significantly associated with mortality (p = 0.0472) [15]. The current study also showed similar results with eosinopenia (OR 3.373, p = 0.024, CI: 1.177-9.669). Marti et al. in Spain (2022) conducted a retrospective cohort study that showed similar results, with 36.8% mortality in the continuous positive airway pressure (CPAP) group and 60.8% mortality in the NIV group. Patients who underwent non-invasive ventilation (NIV) had a higher risk of intubation or death at 28 days (p = 0.004) [16]. Xia et al. in 2021 conducted a multicenter retrospective cohort study that showed COVID-positive patients in Quebec and Ontario who had a shorter length of hospital stay were associated with higher mortality (p-value < 0.01) [17]. Similar results were also found in our study: 0 to seven days of hospital stay had a significant association with mortality (24%, p<0.001, OR 6.531). The study on frailty during the COVID-19 pandemic in Japan in February 2023 revealed a significant increase in frailty prevalence among older adults from 2020 to 2021, compared to pre-pandemic years (2017-2019). The proportion of robust individuals decreased, while both pre-frailty and frailty rates rose. This progression was multifaceted, with declines observed in physical function, daily living activities, oral function, and mental health. The stay-at-home policy and reduced social interaction during the pandemic further contributed to the deterioration, particularly in areas like outdoor activity and social participation. These findings highlight the need for continued monitoring and interventions to address frailty in older adults during and beyond the pandemic [18].

A review article by König et al. highlighted a significant increase in frailty among older adults following the COVID-19 pandemic, referring to this as a potential "frailty wave." Both direct viral effects of COVID-19 and indirect factors, such as lockdown-induced physical inactivity and social isolation, contributed to this rise in frailty. Many older adults experienced a decline in physical and mental functions, with symptoms like fatigue, sarcopenia, and cognitive decline becoming common [19]. In this prospective study, we observed that frailty can contribute to mortality. This might be because mostly frail elderly patients are more prone to infections because of their reduced immune response, and it can lead to worse health outcomes.

Strengths and limitations of this study

The main strength of this study is that the Indian geriatric COVID population was taken into account, which was not done previously. Frailty being one of the predictors of mortality was assessed in all the geriatric COVID-positive patients. Our study also takes into consideration other risk factors like comorbidities, laboratory parameters, and length of stay in the hospital. The limitation of this study was that it was done in a single tertiary care hospital over a small cohort. Cognition and depression could not be assessed in most of the elderly during admission, as they were delirious due to multiple causes. Most patients were obtained do not intubate (DNI) and do not resuscitate (DNR), and those patients received only BiPAP as maximum support. Future follow-up studies are necessary to identify the long-term impact of COVID-19 on frailty.

## Conclusions

This study highlights that frailty significantly increases mortality risk in elderly COVID-19 patients, with CKD and malignancy identified as critical comorbidities. Parameters such as leukocytosis, neutrophilia, anemia, hypoalbuminemia, and elevated liver enzymes were identified as potential mortality indicators. Additionally, patients requiring oxygen support, BiPAP, mechanical ventilation, and those with shorter hospital stays faced higher mortality risks, emphasizing the need for targeted management strategies in this vulnerable group. This further emphasized the need for a comprehensive approach to patient evaluation. Regular frailty assessments are essential in the post-COVID sequelae for early identification of health declines, allowing for timely preventive measures such as vaccination, lifestyle modifications, or targeted screening. These assessments help clinicians address potential complications early and improve long-term outcomes for patients recovering from COVID-19. By integrating frailty assessments with key laboratory markers and respiratory support requirements, clinicians can gain a more holistic view of a patient's health, leading to more informed decision-making and potentially improved patient care.

## References

[1] Ciarambino T, Crispino P, Minervini G, Giordano M (2023). COVID-19 and frailty. Vaccines (Basel).

[2] Wiersinga WJ, Rhodes A, Cheng AC, Peacock SJ, Prescott HC (2020). Pathophysiology, transmission, diagnosis, and treatment of coronavirus disease 2019 (COVID-19): a review. JAMA.

[3] Hewitt J, Carter B, Vilches-Moraga A (2020). The effect of frailty on survival in patients with COVID-19 (COPE): a multicentre, European, observational cohort study. Lancet Public Health.

[4] Popadic V, Klasnja S, Milic N (2021). Predictors of mortality in critically ill COVID-19 patients demanding high oxygen flow: a thin line between inflammation, cytokine storm, and coagulopathy. Oxid Med Cell Longev.

[5] Chen Z, Xu W, Ma W (2021). Clinical laboratory evaluation of COVID-19. Clin Chim Acta.

[6] Moorhouse P, Rockwood K (2012). Frailty and its quantitative clinical evaluation. J R Coll Physicians Edinb.

[7] Rottler M, Ocskay K, Sipos Z (2022). Clinical Frailty Scale (CFS) indicated frailty is associated with increased in-hospital and 30-day mortality in COVID-19 patients: a systematic review and meta-analysis. Ann Intensive Care.

[8] Sablerolles RS, Lafeber M, van Kempen JA (2021). Association between Clinical Frailty Scale score and hospital mortality in adult patients with COVID-19 (COMET): an international, multicentre, retrospective, observational cohort study. Lancet Healthy Longev.

[9] Thakur B, Dubey P, Benitez J (2021). A systematic review and meta-analysis of geographic differences in comorbidities and associated severity and mortality among individuals with COVID-19. Sci Rep.

[10] Fang X, Li S, Yu H (2020). Epidemiological, comorbidity factors with severity and prognosis of COVID-19: a systematic review and meta-analysis. Aging (Albany NY).

[11] Klein IA, Rosenberg SM, Reynolds KL (2021). Impact of cancer history on outcomes among hospitalized patients with COVID-19. Oncologist.

[12] Huang J, Cheng A, Kumar R, Fang Y, Chen G, Zhu Y, Lin S (2020). Hypoalbuminemia predicts the outcome of COVID-19 independent of age and co-morbidity. J Med Virol.

[13] Arnau-Barrés I, Pascual-Dapena A, López-Montesinos I (2021). Severe hypoalbuminemia at admission is strongly associated with worse prognosis in older adults with SARS-CoV-2 infection. J Clin Med.

[14] Suresh Kumar VC, Harne PS, Mukherjee S (2020). Transaminitis is an indicator of mortality in patients with COVID-19: a retrospective cohort study. World J Hepatol.

[15] Yan B, Yang J, Xie Y, Tang X (2021). Relationship between blood eosinophil levels and COVID-19 mortality. World Allergy Organ J.

[16] Marti S, Carsin AE, Sampol J (2022). Higher mortality and intubation rate in COVID-19 patients treated with noninvasive ventilation compared with high-flow oxygen or CPAP. Sci Rep.

[17] König M, Gollasch M, Komleva Y (2023). Frailty after COVID-19: the wave after?. Aging Med (Milton).

[18] Xia Y, Ma H, Buckeridge DL (2022). Mortality trends and length of stays among hospitalized patients with COVID-19 in Ontario and Québec (Canada): a population-based cohort study of the first three epidemic waves. Int J Infect Dis.

[19] Hirose T, Sawaya Y, Ishizaka M, Hashimoto N, Kubo A, Urano T (2023). Frailty under COVID-19 pandemic in Japan: changes in prevalence of frailty from 2017 to 2021. J Am Geriatr Soc.

